# Editorial: Oxidative stress in breast cancer

**DOI:** 10.3389/fonc.2025.1661039

**Published:** 2025-09-16

**Authors:** Ronny Westerman, Zhenbao Liu, Rohimah Mohamud, Ali Mussa

**Affiliations:** ^1^ Federal Institute for Population Research Das Bundesinstitut für Bevölkerungsforschung (BiB), Wiesbaden, Germany; ^2^ Department of Pharmaceutics, Xiangya School of Pharmaceutical Sciences, Central South University, Changsha, Hunan, China; ^3^ Department of Immunology, School of Medical Sciences, Universiti Sains Malaysia, Kubang Kerian, Kelantan, Malaysia; ^4^ Department of Biology, Faculty of Education, Omdurman Islamic University, Omdurman, Sudan

**Keywords:** oxidative stress, ROS - reactive oxygen species, breast cancer, vitamin C, antioxidant, pro-oxidant

**Figure 1:**
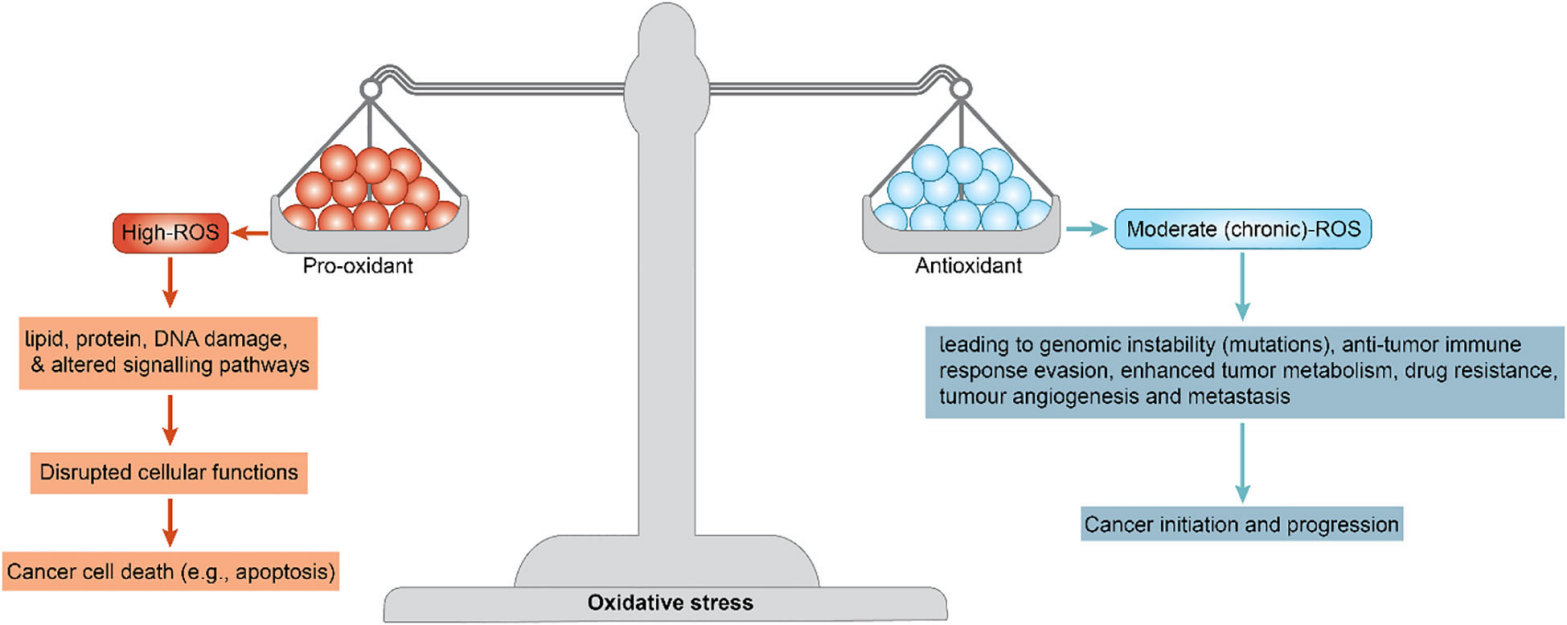
The oxidative stress process in breast cancer.

Breast cancer is one of the most prevalent and challenging malignancies worldwide. In addition to well-characterized genetic and hormonal drivers, there is increasing recognition that oxidative stress plays a pivotal role in breast cancer biology (1). Excess reactive oxygen species (ROS) can cause lipid, protein, and DNA damage. It can also alter signaling pathways and disrupt cellular functions, leading to cancer cell death (e.g., apoptosis) via oxidative stress imbalance. Moderate (chronic) ROS levels can contribute to cancer initiation, leading to genomic instability (mutations), an anti-tumor immune response, enhanced tumor metabolism, drug resistance, tumor angiogenesis, and metastasis (**Figure 1**) (2, 3).

Oxidative stress may be considered to play a “double-edged sword role”; while chronic ROS exposure can promote malignancy in cancers, acute or high ROS bursts can be cytotoxic to cancer cells and are exploited by many therapies.

This Research Topic’s five papers illustrate diverse facets of oxidative stress in breast cancer ―from immune modulation and drug resistance to therapy side effects and biomarkers―and are summarized below.

A review, entitled From Immunogenic Cell Death to Immunogenic Modulation: Select Chemotherapy Regimens Induce a Spectrum of Immune-enhancing Activities in the Tumor Microenvironment <investigated the mechanisms by which specific chemotherapeutic agents promote anti-tumor immunity through the induction of immunogenic cell death (ICD) or immunogenic modulation in cancer cells. Fabian et al. propose the novel concept of “immunogenic cell stress” as a pivotal paradigm within this field of enquiry. The authors posit that this oxidative immunostimulation can be leveraged by combining standard chemotherapies (e.g., anthracyclines) with immunotherapy to capitalize on immunogenic cell stress. The objective of this approach is to convert “cold” tumors into “hot” ones from an immunological perspective by leveraging therapy-induced oxidative stress.

The study *Integrative Dissection of 5-hydroxytryptamine Receptors (5-HTRs; HTR1A, HTR1B, HTR2A, HTR2B, HTR2C, HTR4, and HTR7)-related Signature in the Prognosis and Immune Microenvironment of Breast Cancer* examined how chronic psychological stress (depression) might influence breast cancer via 5-hydroxytryptamine (5-HT) receptors. Among the receptors, the expression of HTR2A/2B exhibited a positive correlation with the infiltration of immune cells, including CD8+ T cells and macrophages. Moreover, the inhibition of HTR2A expression may reduce CD8+ T cell proliferation while promoting the invasion and metastasis of breast cancer cells in zebrafish and mouse models. The findings indicate that HTR2A/2B may significantly modulate the tumor microenvironment, thereby influencing cancer progression. These findings also support the hypothesis that depression is associated with worse breast cancer outcomes, potentially through stress-related inflammation and oxidative stress.

Zhan et al. identified a prognostic tumor gene signature centered on HTR2A and HTR2B (serotonin receptor genes). HTR2A/B expression was reduced in more aggressive cancers, and higher levels of these receptors correlated with better recurrence-free survival and greater T-cell infiltration in tumors. The loss of HTR2A/B signaling resulted in weaker immunity and more metastasis in experimental models. While the study did not measure oxidative stress directly, the results suggest that robust serotonin signaling may counteract the harmful effects of chronic stress, which often elevates oxidative stress and suppresses immunity.

Clinically, this implies that treating depression or modulating 5-HT pathways could benefit breast cancer patients by preserving an effective anti-tumor immune environment.

The mini review *Targeting Ferroptosis: A Promising Strategy to Overcome Drug Resistance in Breast Cancer* emphasized the pathway of ferroptosis as a strategy to overcome breast cancer drug resistance. Ferroptosis is a form of cell death driven by iron-dependent lipid peroxidation and toxic ROS accumulation. Many drug-resistant cancer cells evade apoptosis but remain susceptible to this oxidative form of death. Peng et al. note that drug-resistant breast tumor cells often upregulate antioxidant systems (glutathione, GPX4, etc.) to avoid ferroptosis. Disabling these defenses can re-sensitize tumors: for example, blocking cystine uptake or inhibiting GPX4 unleashes lethal lipid peroxidation in chemo-resistant cells. Inducing ferroptosis in models can eliminate therapy-resistant cells and reduce tumors. Therapies that trigger ferroptosis (e.g. small-molecule inhibitors or nanoparticle-delivered agents) could be combined with standard treatments to eradicate residual disease. Selective induction of ferroptosis in tumors is challenging, but this strategy exploits oxidative stress to circumvent drug resistance.

The study *Effect of Fangxia-Dihuang Decoction on Doxorubicin-induced Cognitive Impairment in Breast Cancer Animal Model* addressed the issue of chemo brain in a breast cancer mouse model. Doxorubicin (DOX) caused cognitive deficits in mice, with elevated oxidative stress markers and inflammatory cytokines in the brain. Wang et al. tested Fangxia-Dihuang Decoction (FXDH), a traditional herbal medicine, as a neuroprotective treatment during DOX treatment. Mice receiving FXDH performed better on memory tests and had lower levels of lipid peroxidation and inflammation compared to DOX-only controls.

FXDH boosted antioxidant defenses (e.g. GSH, SOD) and reduced neuroinflammation in the hippocampus, preventing DOX-induced neuronal damage. Oxidative stress and inflammation are key drivers of chemo brain and counteracting them can preserve cognitive function. Antioxidant and anti-inflammatory supplements (like components of FXDH) could mitigate cognitive side effects of chemotherapy. Such interventions might improve patients’ quality of life and treatment tolerance by reducing oxidative damage in the nervous system.

The study *Association between Neutrophil-lymphocyte Ratio and Female Breast Cancer: An observational study from the NHANES 2001–2018* investigated the neutrophil-to-lymphocyte ratio (NLR), a systemic inflammation marker, in relation to breast cancer. Xiong et al. found that higher NLRs were associated with greater odds of breast cancer. Women in the highest NLR quartile had approximately 67% higher odds of breast cancer than those in the lowest quartile (OR ~1.67). This trend held across subgroups and was confirmed in an external cohort, indicating a robust link between NLR and breast cancer. An elevated NLR suggests an immune system biased toward a neutrophil-driven inflammatory response. Neutrophils release ROS and other mediators, creating a pro-oxidative, tumor-promoting environment. This supports the idea that chronic inflammation and oxidative stress can facilitate cancer development. NLR is an easy, cost-effective biomarker that could help identify individuals with high inflammatory status for monitoring or preventive measures.

This comprehensive body of research demonstrates that oxidative stress plays a dual role in the development of breast cancer. While excess ROS and inflammation can drive tumor growth and spread and treatment resistance, these pathways can also be targeted for therapy. Strategies to mitigate oxidative stress and inflammation, such as stress relief and tissue preservation, can improve outcomes and quality of life.

Strategies to augment oxidative stress within tumors, such as inducing ferroptosis or leveraging immunogenic cell stress, have the potential to enhance cell killing. The potential for reducing oxidative damage while exploiting tumor-specific vulnerabilities is significant.

Future therapeutic interventions will integrate antioxidant and pro-oxidant strategies to boost treatment efficacy while minimizing damage to normal cells.
